# Association Between Maladaptive Eating Behaviors Among Black Women and Vicarious Racial Discrimination Following a High-Profile Event

**DOI:** 10.1007/s40615-024-01994-2

**Published:** 2024-04-05

**Authors:** Kristal Lyn Brown, Amie F. Bettencourt, Anika L. Hines, Lisa A. Cooper, Kimberly A. Gudzune

**Affiliations:** 1 Department of Medicine, School of Medicine, Johns Hopkins University, Baltimore, MD USA; 2 Department of Psychiatry and Behavioral Sciences, Johns Hopkins School of Medicine, Baltimore, MD USA; 3 Department of Health Policy, Virginia Commonwealth University School of Population Health, Richmond, VA USA; 4 Department of Creative Arts Therapies, College of Nursing and Health Professions, Drexel University, Philadelphia, PA 19104 USA

**Keywords:** Racial discrimination, Civil unrest, Disordered eating, Emotional eating, Cognitive restraint, Black women

## Abstract

**Objective:**

Evidence suggests that racial discrimination causes stress among non-Hispanic Black women, and some Black women may cope with exposure to vicarious racial discrimination by engaging in maladaptive eating behaviors.

**Methods:**

We examined eating behaviors among Black women (*N* = 254) before and after Freddie Gray’s death while in police custody. Maladaptive eating behaviors were assessed using the three-factor eating questionnaire. Our independent variables included the following: (1) time period and (2) geographic proximity to the event. Three two-way analysis of covariance tests were conducted to assess potential effects of geographic proximity (close, distant), time period in relation to unrest (before, after unrest), and their interaction on emotional eating, uncontrolled eating, and cognitive restraint controlling for participant age.

**Results:**

There was a statistically significant main effect of proximity to the unrest on emotional eating, *F* (1, 252) = 5.64, *p* = .018, and partial η2 = .022 such that women living in close geographic proximity to the unrest reported higher mean levels of emotional eating as compared to those living more distant to the unrest. There was also a borderline statistically significant interaction between geographic proximity and time period on cognitive restraint, *F* (1, 252) = 3.89, *p* = .050, and partial η2 = .015.

**Conclusion:**

Our study found a relationship between vicarious racial discrimination and maladaptive eating behaviors among Black women. Future work should examine stress related to vicarious racial discrimination and maladaptive eating behaviors longitudinally.

**Supplementary Information:**

The online version contains supplementary material available at 10.1007/s40615-024-01994-2.

## Introduction

A growing body of literature suggests that racial discrimination is linked to psychological and physiological stress [1–3]. Non-Hispanic Black women engage in a variety of coping strategies to endure exposure to racial discrimination to include but not limited to avoidance, social support, religious/spiritual-based activities, and other covert strategies [4]. For some, coping behaviors may also include maladaptive eating behaviors such as emotional and uncontrolled eating [5, 6]. Given the prevalence of maladaptive eating behaviors among Black women and its association with other risk factors [7, 8], it is critical to examine the potential contribution of racial discrimination to this disparity.

According to the environmental affordances (EA) model [9], an individual’s environment can induce stress, but there are also affordances or stress-reducers which can be used as coping strategies. The EA model also asserts that all things (human and otherwise) typically move to engage in self-regulatory coping strategies that will immediately reduce psychological/physiological stress and that factors such as race/gender could influence behaviors [9]. In theory, some Black women may cope with racial discrimination by engaging in maladaptive eating behaviors [10] For example, Black women who participated in an ecological momentary assessment study reported an increase in disordered eating behaviors following experiences with racial discrimination [5]. Of note, the majority of these experiences were vicarious [5]—meaning these experiences were related to negative incidents that participants witnessed happening to others because of their race (not direct experiences themselves). Additionally, a recent systematic review found racial discrimination negatively impacts eating behaviors, including loss of control eating, binge eating, over snacking, and restraint [11]. These studies included both Black and White individuals; however, the majority (63%) were Black/African American samples only. One study found a relation between racial discrimination and binge eating among Black women only such that Black women endorsed greater binge eating. In addition, a recent study conducted by Hoggard and colleagues found a positive association between racism and emotional eating among a sample of Black adults [12].

For Black women, vicarious racial discrimination may occur when they observe racial discrimination or violence towards other members of the Black community [13]—this includes family, friends, colleagues, neighbors, and/or individuals that are not personally known to the individual. Importantly, vicarious racial discrimination is typically reported more frequently compared to personal racial discrimination—particularly among women [14, 15]. Over the years, police violence has been commonplace, with multiple Black individuals dying at the hands of police officers [16]. Witnessing police violence is one way in which individuals could be exposed to racial discrimination vicariously. Neighbors and bystanders are often subjected to emotional and psychological trauma from witnessing racial discrimination via acts of police brutality. In recent years, such events have garnered significant media attention [16, 17] with periods of protest, riots, and civil unrest following many events. A study conducted by Hines and colleagues [18, 19] found a relation between proximity to civil unrest and depressive symptoms among Black individuals living in two low-income neighborhoods. Similarly, participants in another study reported greater maternal depressive symptoms following civil unrest in their community as compared to before the event [20].

With the increase in video footage and various media outlets [16, 17] (e.g., Twitter, TV, and TikTok), the reach and potential physical and mental health impact of vicarious racial discrimination has continued to rise [18, 19]—which means those who are not present at the scene of the event could still be exposed to racial discrimination and subsequent harm vicariously. For example, a study conducted by Garrick and colleagues found a significant relation between police violence and vicarious trauma from viewing police brutality videos [21]. Black women in this same study were impacted more than Black men [21]. However, few studies have examined whether such acute events of vicarious racial discrimination influence maladaptive eating habits. Notably, social media is not the only way to be exposed to racial discrimination vicariously. For example, witnessing a discriminatory event in your neighborhood or while dining at a restaurant or traveling could also lead to vicarious exposures. Further, listening to the lived experiences of someone else could have a similar impact.

Regardless of how racial discrimination is experienced, exposure could lead to race-based traumatic stress (RBTS) [22]. RBTS is the psychological and emotional response to racism/racial discrimination [22]. RBTS is quite complex and linked to both poor mental and physical health outcomes [23]. Importantly, every discriminatory event might not trigger a traumatic response [22]; however, exposure to racial discrimination particularly cumulatively increases one’s risk [22, 23]. Additionally, factors such as racial identity and worldview could moderate the perception of racial discrimination, which could also influence response [22].

To that end, we conducted a secondary data analysis, drawing upon data collected from a community-based sample in our parent study, Communities CARING. We examined the influence of vicarious racial discrimination by comparing differences before and after a high-profile event involving police officers in Baltimore, MD. We hypothesized that reports of maladaptive eating would be higher post-unrest compared to pre-unrest. We further hypothesized that participants residing in the neighborhood where the event occurred would endorse greater maladaptive eating behaviors compared to individuals residing in a similar neighborhood that was more distant to the event.

## Methods

### Description of the Event

Trigger warning: Please be advised the that following paragraph includes content that is disturbing and may be traumatizing for some readers.

On April 12, 2015, Freddie Gray, a 25-year-old Black man residing in West Baltimore, was arrested by White police officers for carrying a knife. Shortly after his arrest, his neck was broken, and spinal cord almost completely severed while being transported in a police van. Mr. Gray died from his injuries a week later. Release of video footage of the arrest and bystander testimonies detailing the unnecessary use of extreme force against a young Black man garnered national media attention and spurred subsequent protests, civil unrest, and riots. The height of the unrest and media attention occurred after Mr. Gray’s funeral on April 27, 2015—many police officers were injured, and nearly 150 cars and multiple buildings were burned. Subsequently, the entire city of Baltimore was given a 10 pm curfew, and the National Guard was activated to assist with maintaining the peace after riots broke out.

### Parent Study, Participants, and Procedures

Prior to and immediately following the unrest, researchers were collecting cross-sectional survey data for the Communities CARING study. This study collected information on health behaviors among residents in two public housing developments—one in West Baltimore (Gilmor Homes) and the other in East Baltimore (Latrobe Homes). Mr. Gray’s arrest occurred in Gilmor Homes. Households were randomly selected to participate, and up to four adults in each household could enroll (total *N* = 358). All addresses were randomly selected at the beginning of the study, and no new addresses were added. Data was collected from August 2014 to August 2015, spanning the death of Mr. Gray and the subsequent media attention and unrest. All addresses were randomly selected at the beginning of the study, and no new addresses were added. All participants received the same incentives regardless of being in the pre-post group. The study protocol, including all procedures and recruitment materials were approved by the Johns Hopkins University Institutional Review Board.

### Study Design

We conducted a secondary analysis of cross-sectional data collected as part of the Communities CARING study both before and after this high-profile event. Given our research question, we limited the analytic sample to Black women (*N* = 257) for these secondary analyses.

### Measures

#### Maladaptive Eating (Dependent Variables)

Maladaptive eating behaviors were assessed using three subscales—cognitive restraint, uncontrolled eating, and emotional eating—from the previously validated three-factor eating questionnaire [24]. While uncontrolled and emotional eating are typically associated with stress response/coping, we also included cognitive restraint due to the exploratory nature of this study. Cognitive restraint examined purposeful food restriction for weight loss and an example item is “I consciously hold back at meals in order not to weight gain.” Uncontrolled eating examined experiencing a loss of control over food intake, thus leading to greater than usual consumption and an example item is “Sometimes when I start eating, I just can’t seem to stop.” And emotional eating examined the inability to abstain from emotional cues and an example item is “When I feel blue, I often overeat.” For each participant, we calculated a score for each subscale as recommended per the instrument (higher scores indicate greater degree of the specific eating behavior). Given the short time course, we did not examine differences in body mass index (BMI)—changes and conditions that typically require greater duration of time to occur.

#### Time Period and Geographic Proximity (Independent Variables)

Our independent variables included the following: (1) time period and (2) geographic proximity to the event. We used an approach previously used in a similar study [25]. For time period, we defined “before event” as survey participants who completed interviews before April 27th and surveys after this date as “after event.” For geographic proximity, we defined participants in Gilmor Homes as “close” and Latrobe Homes as “distant,” given that Mr. Gray’s arrest took place in Gilmor Homes.

#### Covariates

We considered age as a covariate considering existing research suggesting that eating behaviors vary by age [26, 27]. Furthermore, the response to vicarious racial discrimination and trauma may be different based on age/life stage.

### Statistical Analyses

Descriptive statistics were used to characterize the sample including demographics, health status, and perceived neighborhood factors among our sample. Bivariate analyses, including independent samples *t*-tests for continuous variables and chi-square analyses for categorical variables, were conducted to compare sample characteristics by geographic proximity to the unrest alone and by time period within the context of geographic proximity to the unrest. Using the univariate general linear model procedure in SPSS 28 (IBM Corp, 2021), three two-way analysis of covariance (ANCOVA) tests were conducted to assess the potential effects of geographic proximity (close, distant), time period in relation to unrest (before April 27, after April 27), and the interaction between these factors on the three eating behaviors (emotional eating, uncontrolled eating, cognitive restraint) controlling for participant age at the time of the study. Post hoc simple main effects tests were conducted to explore significant interactions. Before conducting each ANCOVA, the data were examined for the assumptions of the analysis, including linearity, homogeneity of regression slopes, homogeneity of variance, and normality. All assumptions of two-way ANCOVA were met for the cognitive restraint scale, and all but one was met for the emotional eating and uncontrolled eating scales. Specifically, the assumption of normality was violated for the emotional eating and uncontrolled eating scales due to a pile up of scores at or near 0. Despite this violation, we felt comfortable moving forward with the analysis because ANCOVA is relatively robust to departures from normality.

## Results

Characteristics for the total sample are reported in Table 1. On average, the sample was 42.6 years old (SD = 13.2), and 95.3% were single. A majority of the sample had achieved a high school diploma or greater (65.3%) and were employed (63.8%). On average, the sample had lived in their neighborhoods for 7.0 years (SD = 7.3) and a majority reported concerns about neighborhood crime during the day and night. Mean BMI for the sample met criteria for obesity (*M* = 32.8, SD = 10.2). History of cardiovascular diseases (myocardial infarction and congestive heart failure) were uncommon. A majority of the sample were currently smokers.

**Table 1:** Characteristics of the sample overall and by geographic proximity* to the unrest

Characteristic	Overall sample, *N* = 257	Close to unrest, *n* = 134	Distant from unrest, *n* = 123	Chi-square/*t*-statistic	*p*-value
Age in years; mean (SD)	42.6 (13.2)	42.3 (12.5)	42.9 (13.9)	− 0.38	0.706
Education				4.77	0.092
< High school graduate	89 (34.6)	53 (39.6)	36 (29.3)		
High school graduate	98 (38.1)	43 (32.1)	55 (44.7)		
> High school graduate	70 (27.2)	38 (28.4)	32 (26.0)		
Marital status				1.06	0.302
Married/domestic partnership	12 (4.7)	8 (6.0)	4 (3.3)		
Not married or partnered	245 (95.3)	126 (94.0)	119 (96.7)		
Employment status				0.15	0.695
Employed	164 (63.8)	50 (37.3)	43 (35.0)		
Unemployed	93 (36.2)	84 (62.7)	80 (65.0)		
Insurance status				1.20	0.273
Has health insurance	253 (98.4)	133 (99.3)	120 (97.6)		
No health insurance	4 (1.6)	1 (0.7)	3 (2.4)		
# years in living environment; mean (SD)	7.0 (7.3)	6.6 (7.0)	7.3 (7.8)	− 0.81	0.420
Neighborhood attributes^1^					
Perceived daytime crime	170 (66.1)	103 (76.9)	67 (54.5)	14.36	< 0.001
Perceived nighttime crime	212 (82.5)	119 (88.8)	93 (75.6)	7.73	0.005
Social cohesion; mean (SD)	3.0 (0.5)	3.0 (0.6)	3.0 (0.5)	0.97	0.336
Depressive symptoms	82 (31.9)	45 (33.6)	37 (30.1)	0.36	0.548
History of myocardial infarction	13 (5.1)	9 (6.7)	4 (3.3)	1.60	0.206
History of congestive heart failure	10 (3.9)	5 (3.7)	5 (4.1)	0.02	0.890
Current smoker				3.82	0.051
Yes	158 (61.5)	90 (67.2)	68 (55.3)		
No	99 (38.5)	44 (32.8)	55 (44.7)		
BMI; mean (SD)	32.8 (10.2)	31.2 (9.4)	34.6 (10.9)	− 2.73	0.007
Eating-related variables^2^					
Emotional eating; mean (SD)	24.6 (22.6)	27.7 (23.5)	21.3 (21.1)	2.31	0.022
Uncontrolled eating; mean (SD)	22.4 (16.1)	23.8 (15.4)	20.9 (16.7)	1.46	0.145
Cognitive restraint; mean (SD)	27.5 (16.8)	28.0 (16.6)	27.1 (17.2)	0.42	0.672

Data are expressed as *n* (%) unless otherwise noted. For daytime and nighttime crime, dichotomous variables were created such that somewhat agree or strongly agree with statements about the crime rate making it unsafe to go on walks in their neighborhood was scored as 1 and all other responses were scored as 0. *Individuals residing in Gilmor Homes in West Baltimore were considered “close to unrest” and those residing in Latrobe Homes in East Baltimore were considered “distant from the unrest”

^1^Cohen DA, Finch BK, Bower A, Sastry N. Collective efficacy and obesity: the potential influence of social factors on health. *Social Science & Medicine.* 2006;62(3):769–778

^2^de Lauzon B, Romon M, Deschamps V, et al. The Three-Factor Eating Questionnaire-R18 is able to distinguish among different eating patterns in a general population. *Journal of Nutrition.* 2004;134(9):2372–2380

Table 1 also displays comparisons of sample characteristics by geographic proximity to the unrest. Overall, the two neighborhoods were similar on most of the characteristics examined. However, there were statistically significant differences on measures of daytime and nighttime crime, BMI, and emotional eating. Specifically, a larger proportion of individuals residing in the neighborhood that was in closer geographic proximity to the unrest reported feeling unsafe during the day and at night as compared to those residing in the neighborhood that was more distant from the unrest. Those residing in the neighborhood in closer proximity to the unrest also reported higher mean levels of emotional eating and a larger proportion of individuals in this neighborhood reported smoking as compared to those residing in the neighborhood that was more distant from the unrest.

Table 2 shows comparisons on key sample characteristics by time period in relation to the unrest within the context of geographic proximity to the unrest. Overall, study participants in the two neighborhoods were similar on all variables regardless of whether they participated in the study before or after the unrest with one exception. Specifically, individuals residing in the neighborhood in close proximity to the unrest who completed study measures after the unrest reported significantly higher levels of cognitive restraint as compared to those within the same neighborhood who completed study measures before the unrest.

**Table 2:** Characteristics of the sample by geographic proximity* and time period in relation to the unrest

Characteristic	Close to unrest, *n* = 134			Distant from unrest, *n* = 123		
	Before April 27, *n* = 74	After April 27, *n* = 60	*p*-value	Before April 27, *n* = 82	After April 27, *n* = 41	*p*-value
Age in years; mean (SD)	43.5 (12.0)	40.9 (13.0)	0.232	44.0 (13.8)	40.8 (14.0)	0.219
Neighborhood attributes^1^						
Perceived daytime crime; *n* (%)	53 (71.6)	50 (83.3)	0.110	42 (51.2)	25 (61.0)	0.306
Perceived nighttime crime; *n* (%)	65 (87.8)	54 (90.0)	0.693	62 (75.6)	31 (75.6)	0.99
Social cohesion; mean (SD)	3.0 (0.5)	3.0 (0.6)	0.747	3.0 (0.5)	2.9 (0.5)	0.139
Depressive symptoms; *n* (%)	21 (28.4)	24 (40.0)	0.157	28 (34.1)	9 (22.0)	0.164
History of myocardial infarction	4 (5.4)	5 (8.3)	0.501	4 (4.9)	0 (0.0)	0.150
History of congestive heart failure	3 (4.1)	2 (3.3)	0.827	5 (6.1)	0 (0.0)	0.106
Current smoker			0.795			0.608
Yes	49 (66.2)	41 (68.3)		44 (53.7)	24 (58.5)	
No	25 (33.8)	19 (31.7)		38 (46.3)	17 (41.5)	
BMI; mean (SD)	30.9 (10.0)	31.5 (8.6)	0.716	33.3 (11.1)	32.2 (10.0)	0.345
Eating-related variables^2^						
Emotional eating; mean (SD)	27.7 (23.4)	27.8 (23.9)	0.985	19.5 (19.3)	24.4 (22.4)	0.516
Uncontrolled eating; mean (SD)	24.1 (16.0)	23.4 (14.8)	0.793	21.0 (17.6)	20.6 (14.7)	0.914
Cognitive restraint; mean (SD)	24.8 (15.4)	31.8 (17.3)	0.014	27.6 (17.3)	26.0 (17.3)	0.634

For daytime and nighttime crime, dichotomous variables were created such that somewhat agree or strongly agree with statements about the crime rate making it unsafe to go on walks in their neighborhood was scored as 1 and all other responses were scored as 0. *p*-values for continuous variable comparisons are based on independent samples *t*-tests. *p*-values for categorical variable comparisons are based on chi-square analyses. *Individuals residing in Gilmor Homes in West Baltimore were considered “close to unrest,” and those residing in Latrobe Homes in East Baltimore were considered “distant from the unrest”

^1^Cohen DA, Finch BK, Bower A, Sastry N. Collective efficacy and obesity: the potential influence of social factors on health. *Social Science & Medicine.* 2006;62(3):769–778

^2^de Lauzon B, Romon M, Deschamps V, et al. The Three-Factor Eating Questionnaire-R18 is able to distinguish among different eating patterns in a general population. *Journal of Nutrition.* 2004;134(9):2372–2380

Table 3 displays results of the three ANCOVA models controlling for age. There was a statistically significant main effect of proximity to the unrest on emotional eating, *F* (1, 252) = 5.64, *p* = 0.018, and partial η2 = 0.022 such that women living in close geographic proximity to the unrest reported higher mean levels of emotional eating as compared to those living in the neighborhood more distant to the unrest. There were no significant differences by time period in relation to the unrest on emotional eating. There were also no significant differences by geographic proximity or time period in relation to unrest on uncontrolled eating. There was a borderline statistically significant interaction between geographic proximity and time period in relation to the unrest on cognitive restraint, *F* (1, 252) = 3.89, *p* = 0.050, and partial η2 = 0.015. Probing of the simple main effects within the interaction revealed significant differences in mean levels of cognitive restraint before versus after the unrest within the neighborhood in close geographic proximity to the unrest, *F* (1, 252) = 5.80, *p* = 0.017, and partial η2 = 0.023; among women living in close proximity to the unrest, those who participated in the study after the unrest reported higher levels of cognitive restraint than those who participated in the study prior to the unrest.

**Table 3:** General linear models examining associations between proximity and time in relation to the unrest as predictors of disordered eating variables

Characteristic	Emotional eating			Uncontrolled eating			Cognitive restraint		
	Adj. mean (SE)	*F*	*p*-value	Adj. mean (SE)	*F*	*p*-value	Adj. mean (SE)	*F*	*p*-value
Age in years	42.6	0.12	0.735	42.6	0.79	0.374	42.6	0.05	0.816
Proximity to unrest		5.64	0.018		1.99	0.159		0.50	0.482
Close	27.8 (2.0)			23.7 (1.4)			28.3 (1.5)		
Distant	20.8 (2.2)			20.8 (1.5)			26.8 (1.6)		
Time period related to unrest		0.16	0.688		0.12	0.728		1.61	0.206
Before April 27	24.9 (1.8)			22.6 (1.3)			26.2 (1.4)		
After April 27	23.7 (2.3)			21.9 (1.6)			29.0 (1.7)		
Proximity*time period interaction		0.21	0.644		0.01	0.940		3.89	0.050
Distant, before April 27	22.1 (2.5)			21.1 (1.8)			27.6 (1.9)		
Distant, after April 27	19.7 (3.5)			20.5 (2.5)			26.1 (2.6)		
Close, before April 27	27.7 (2.6)			24.2 (1.9)			24.8 (2.0)		
Close, after April 27	27.8 (2.9)			23.3 (2.1)			31.9 (2.2)		

*Adj. mean* the mean adjusted for the covariate of age, *SE* standard error. Individuals residing in Gilmor Homes in West Baltimore were considered “close to unrest,” and those residing in Latrobe Homes in East Baltimore were considered “distant from the unrest”

## Discussion

Vicarious racial discrimination is a major stressor for Black individuals. One way in which individuals can be exposed to vicarious racial discrimination is by witnessing police violence and subsequent civil unrest. Our study examined the relation between civil unrest due to police violence, proximity to the unrest, and eating behaviors (uncontrolled, emotional, cognitive restraint) among Black women. In the paragraphs to follow, we discuss our findings from this study and the impact of vicarious racial discrimination more broadly.

In our study, emotional eating differed between the two neighborhoods, such that women residing in West Baltimore reported greater emotional eating. Parallel to this, West Baltimore women also perceived greater daytime crime and reported a higher percentage of smoking compared to those in East Baltimore. These findings underscore the idea that women residing in West Baltimore have higher stress and are engaging in multiple coping behaviors—one of which could be emotional eating to cope with stress related to feeling chronically unsafe [28]. The relationship between eating behaviors and stress has been well researched [29–31]. These findings also align with the environmental affordances model [9], which links environmental stressors to self-regulatory behaviors, such as emotional eating—which could help mental health in the short-term, but ultimately could lead to poor physical and mental health outcomes long term [32]. It is also important to emphasize the potential intergenerational effects of emotional eating, specifically among mothers and daughters [33] and those with poor emotional regulation [32]. While it is unclear if intergenerational effects are primarily due to modeling behavior or reinforcement of behaviors [33], the cyclic outcomes are very much the same—a child being introduced to consuming food as a coping mechanism, who eventually grows up to be an adult who might also engage in emotional eating as a self-regulatory practice. Of note, we saw no differences in uncontrolled eating between neighborhoods or time periods. This finding is opposite of much of the stress-based literature in that evidence suggests that greater stress is associated with both emotional and uncontrolled eating [34–36]. Perhaps the timing of when we were able to measure impacted our findings or uncontrolled eating was not as prevalent among our sample in general.

Cognitive restraint also differed between the two neighborhoods such that those residing in closer proximity to the event reported greater cognitive restraint. Cognitive restraint was also higher among individuals that were surveyed after the unrest occurred. Our findings are consistent with general stress-based literature in that evidence suggests some individuals report greater cognitive restraint when they are under increased stress [37]. In theory, women in our study could have been practicing cognitive restraint to prevent eating as a way to cope with stress [37]—more specifically RBTS [22]. Other research has suggested that there is a positive relation between high cognitive restraint and greater urinary cortisol secretion—a known indicator for stress [38, 39]. Further, while evidence is limited, our findings are also consistent with previous literature in that experiences of discrimination could contribute to cognitive restraint [40]—this is particularly true for those who sit at the intersection of more than one identity (e.g., being Black and a woman). Similarly, another study found a link between eating behaviors and ethnic discrimination, such that those reporting greater discrimination also reported higher maladaptive eating behaviors [41]. Admittedly while our findings align with some previous research, results are somewhat surprising considering that emotional and uncontrolled eating is typically known to have a greater association with stress relative to cognitive restraint. These findings highlight the importance of continuing to explore cognitive restraint and stress related to racial discrimination. Future studies should consider using mixed methods to further explore mechanisms that could contribute to cognitive restraint, which might help further explain these findings.

Importantly, vicarious racial discrimination is not limited to police violence. Vicarious racial discrimination could show up in other context such as among colleagues in the workplace or within school settings. For example, Black individuals attending predominately white institutions often report witnessing and/or experiencing racial discrimination [18, 42, 43]. Further, like personal exposure to racial discrimination, experiencing vicarious racial discrimination carries harmful health effects both mentally and physically [44–46].

Findings from our study should be interpreted considering several limitations. First, causality cannot be determined as our study is cross-sectional. Future work should examine the effects of vicarious racial discrimination and eating behaviors among Black women using ecologically valid and longitudinal approaches. Second, the original purpose of this data was to examine health behaviors of public housing residents; therefore, it was not specifically designed for our research question. However, our findings still contribute to the literature by providing a basis to continue exploring the impact of vicarious racial discrimination via unpredictable high-profile events. Third, we did not include depression, or BMI as covariates in our analysis, given the exploratory nature of our study and our sample size. Future work should consider mechanisms that might contribute to racial discrimination and subsequent eating behaviors. Fourth, we cannot fully account for the duration of time pre and post changes due to the unrest during which changes in eating behaviors occurred, which means we are unable to fully disentangle other possible contributing factors. Fifth, we did not examine general stress related to living in each neighborhood which means we were unable to tease out overall stress and stress from the vicarious racial discrimination event specifically. Finally, we did not capture other environmental factors such as structural racism, which contributes to neighborhood food access [47, 48]—this is particularly relevant considering both neighborhoods are classified as food insecure/food desserts [47].

Our study has several strengths including focusing specifically on Black women—a high-risk understudied population. In addition, this study captured data before and after a high-profile discriminatory event. Given the unpredictable nature of acute, high-profile discriminatory events, community data that was collected before and after such an event is highly unique. Finally, this is one of the first studies to examine the relation between eating behaviors and vicarious racial discrimination via police violence within a sample of Black women.

In conclusion, our study found a relation between vicarious racial discrimination and maladaptive eating behaviors among Black women. Specifically, Black women residing in closer proximity to the unrest and who took the survey after the unrest reported higher cognitive restraint. Individuals living in the neighborhood closer to the event also reported higher emotional eating and a greater perception of neighborhood crime compared to the more distal neighborhood. Future work should examine stress related to vicarious racial discrimination and eating behaviors longitudinally—particularly exploring the relation between perceiving your environment as chronically unsafe, RBTS, and maladaptive eating behaviors.

## Supplementary Information

Below is the link to the electronic supplementary material.

Supplementary file1 (DOCX 14 KB)

Supplementary file2 (DOCX 13 KB)
