# Supplementary material for: Association Between Maladaptive Eating Behaviors Among Black Women and Vicarious Racial Discrimination Following a High-Profile Event
Source: J Racial Ethn Health Disparities. 2024 Apr 5;12(3):1621–9. doi: 10.1007/s40615-024-01994-2 (PMC12069145; doi:10.1007/s40615-024-01994-2)
Supplement: Supplementary file 1 — Supplementary file1 (DOCX 14 KB) [file 40615_2024_1994_MOESM1_ESM.docx]

**TFEQ-18**

The Three Factor Eating Questionnaire (TFEQ-18) is an 18-item, self-administered questionnaire. The following are example questions:

1. I deliberately take small helpings as a means of controlling my weight. (Cognitive Restraint)
2. Sometimes when I start eating, I just can’t seem to stop. (Uncontrolled Eating)
3. When I feel blue, I often overeat. (Emotional Eating)

Answer choices:

definitely true (4)

mostly true (3)

mostly false (2)

definitely false (1)
