# Supplementary material for: Association Between Maladaptive Eating Behaviors Among Black Women and Vicarious Racial Discrimination Following a High-Profile Event
Source: J Racial Ethn Health Disparities. 2024 Apr 5;12(3):1621–9. doi: 10.1007/s40615-024-01994-2 (PMC12069145; doi:10.1007/s40615-024-01994-2)
Supplement: Supplementary file 2 — Supplementary file2 (DOCX 13 KB) [file 40615_2024_1994_MOESM2_ESM.docx]

Perception of Neighborhood Crime

1. The crime rate in my neighborhood makes it unsafe to go on walks DURING THE DAY.
2. The crime rate in my neighborhood makes it unsafe to go on walks AT NIGHT.
